# Resolving physical interactions between bacteria and nanotopographies with focused ion beam scanning electron microscopy

**DOI:** 10.1016/j.isci.2021.102818

**Published:** 2021-07-07

**Authors:** Joshua Jenkins, Mohd I. Ishak, Marcus Eales, Ali Gholinia, Satishkumar Kulkarni, Thomas F. Keller, Paul W. May, Angela H. Nobbs, Bo Su

**Affiliations:** 1 Bristol Dental School, University of Bristol, Bristol, UK; 2 Faculty of Engineering Technology, Universiti Malaysia Perlis, Malaysia; 3 School of Materials Science, University of Manchester, Manchester, UK; 4 Deutsches Elektronen-Synchrotron DESY, Notkestraße 85, Hamburg 22607, Germany; 5 Physics Department, University of Hamburg, Hamburg, Germany; 6 School of Chemistry, University of Bristol, Bristol, UK

**Keywords:** Microbiofilms, Surface treatment

## Abstract

To robustly assess the antibacterial mechanisms of nanotopographies, it is critical to analyze the bacteria-nanotopography adhesion interface. Here, we utilize focused ion beam milling combined with scanning electron microscopy to generate three-dimensional reconstructions of *Staphylococcus aureus* or *Escherichia coli* interacting with nanotopographies. For the first time, 3D morphometric analysis has been exploited to quantify the intrinsic contact area between each nanostructure and the bacterial envelope, providing an objective framework from which to derive the possible antibacterial mechanisms of synthetic nanotopographies. Surfaces with nanostructure densities between 36 and 58 per μm^2^ and tip diameters between 27 and 50 nm mediated envelope deformation and penetration, while surfaces with higher nanostructure densities (137 per μm^2^) induced envelope penetration and mechanical rupture, leading to marked reductions in cell volume due to cytosolic leakage. On nanotopographies with densities of 8 per μm^2^ and tip diameters greater than 100 nm, bacteria predominantly adhered between nanostructures, resulting in cell impedance.

## Highlights

• Bacteria-nanotopography interactions can be quantified using FIB-SEM
• Envelope penetration and cell impedance are influenced by nanotopography density
• Low density nanotopographies (8 per μm^2^) mediate cell impedance
• High-density nanotopographies (36–137 per μm^2^) mediate deformation and penetration

## Graphical abstract

**Figure:**
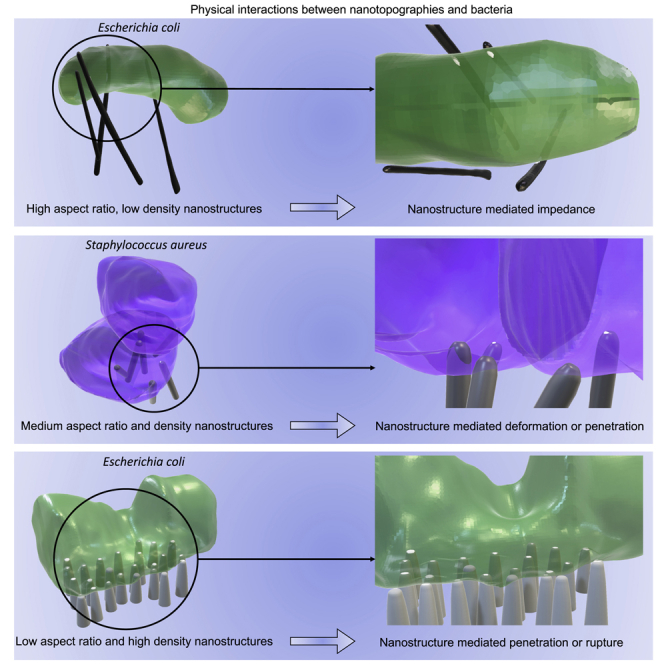

## Introduction

The nanostructures found on cicada and dragonfly wings are widely reported to induce physical stretching of bacterial and fungal cell envelopes upon contact, leading to mechanical rupture, cell lysis, and death (Ivanova et al., 2012; Nowlin et al., 2014). The antimicrobial properties of insect wings have provided significant inspiration for the design of synthetic nanostructures with bactericidal activity (Diu et al., 2014; Dunseath et al., 2019; Fisher et al., 2016; Hazell et al., 2018a, 2018b; Ishak et al., 2020; Jenkins et al., 2020). Determining the underlying mechanisms that drive bacterial cell death on synthetic nanotopographies is crucial, as this will guide the rational design of medical implant surfaces that are resistant to biofilm formation. Several biophysical models have been proposed to explain the mechanisms that drive this antimicrobial phenomenon (Ivanova et al., 2020; Li and Chen, 2016; Linklater et al., 2018; Pogodin et al., 2013; Xue et al., 2015). Alongside the mechanistic theory of contact killing, several biological, chemical and physical factors have been directly linked to promoting nanotopography-mediated antimicrobial activity, including oxidative stress (Jenkins et al., 2020), microbial adhesion force (Bandara et al., 2017; Nowlin et al., 2014), bacterial cell wall thickness (Hasan et al., 2013; Pogodin et al., 2013), chemical composition (Devlin-Mullin et al., 2017; Ewald et al., 2006) and nanotopography geometry (Dewald et al., 2018; Diu et al., 2014; Hazell et al., 2018a, 2018b; Lüdecke et al., 2016; Velic et al., 2019; Watson et al., 2019).

Visualizing the cell-surface interface is crucial for elucidating the antibacterial mechanisms of a nanotopography. Traditionally, these analyzes have been performed using scanning electron microscopy (SEM) (Ivanova et al., 2012; Jenkins et al., 2018). However, this approach cannot resolve cellular ultrastructures such as the bacterial cell wall and membranes. Furthermore, the area of nanotopography that interfaces with the cell envelope is generally concealed from the incident electron beam, thereby restricting the study to surface morphology, mostly in two dimensions. These limitations have prompted alternative techniques that can visualize both nanotopography and bacterial ultrastructure at nanometer resolution; one such technique is focused ion beam scanning electron microscopy (FIB-SEM). Using this approach, biological specimens such as bacteria and fungi can be iteratively cross sectioned and simultaneously imaged, with the potential to generate three-dimensional (3D) volume reconstructions that enable all microbe-nanotopography interactions to be visualized simultaneously (Jenkins et al., 2020).

With precise control over the exact location of surface ablation, FIB-SEM has proved a powerful tool for directly visualizing the contact points between bacteria or fungi and nanotopographies (Bandara et al., 2020; Bhadra et al., 2015; Dewald et al., 2018; Linklater et al, 2017, 2018; Lüdecke et al., 2016). Two main approaches have been utilized to investigate bacteria-nanotopography interactions via FIB-SEM. One method involves generating thin sections, known as lamellae, through bacteria and the underlying nanotopography. Lamellae can then be analyzed by transmission electron microscopy (Jenkins et al., 2018; Linklater et al., 2018). Alternatively, bacteria-nanotopography interactions can be investigated *in situ*, by generating single cross sections through bacterial cells adhered directly to the nanotopography. This approach has been widely used to visualize the interactions between bacteria and individual nanostructures. FIB milling of *Pseudomonas aeruginosa* adhered to cicada wings revealed how natural nanostructures can rupture the bacterial envelope, causing cells to submerge into the nanotopography (Ivanova et al., 2012). Similarly, FIB-SEM analysis of *P. aeruginosa* on dragonfly wing-inspired titanium nanostructures found membrane deformation caused by the energy gain from surface attachment (Bhadra et al., 2015). Initial stretching of *Staphylococcus aureus* and *P. aeruginosa* cell envelopes on black silicon (bSi) nanostructures has also been discovered by FIB-SEM cross-sectional analysis (Linklater et al., 2018). Furthermore, FIB-SEM has identified cytoplasmic leakage from *Staphylococcus epidermidis* cells caused by envelope penetration from spear-like titanium nanostructures (Cao et al., 2018). Most recently, localized envelope deformation and penetration of *Escherichia coli* and *S. aureus* cells incubated on TiO_2_ nanostructures generated by thermal oxidation was identified (Jenkins et al., 2020).

Although single cross sections generated by FIB milling reveal how bacterial envelope morphology changes at the point of nanostructure contact, this approach does not enable the frequency of nanostructure-induced envelope deformation or penetration to be quantified at a single cell level. Furthermore, it does not reveal the surface area of bacterial envelope that is in direct contact with each nanostructure and to what degree this influences the extent of deformation and/or frequency of penetration. To comprehensively quantify these parameters, this study generated four surface types with distinct nanostructure geometries and utilized a slice-by-slice FIB-SEM milling approach to directly visualize the adhesion interface between *S. aureus* or *E. coli* and individual nanostructures. Slice-by-slice FIB-SEM data were then used to generate 3D volume reconstructions of whole bacteria in contact with the underlying nanotopography, enabling all contact points between the bacterial envelope and nanostructures to be resolved simultaneously with nanometer resolution. This approach was previously impossible using conventional 2D imaging tools. Furthermore, advances in 3D analysis software (Cocks et al., 2018; Jorstad et al., 2015) enabled direct quantification of bacteria-nanotopography interactions, including the effective contact surface area between each nanostructure and the bacterial envelope. These analyses demonstrate how this approach can be used to develop an objective framework from which the antibacterial mechanisms of synthetic nanotopographies can be derived.

## Results

### Fabrication and characterization of nanotopographies

**Figure 1:**
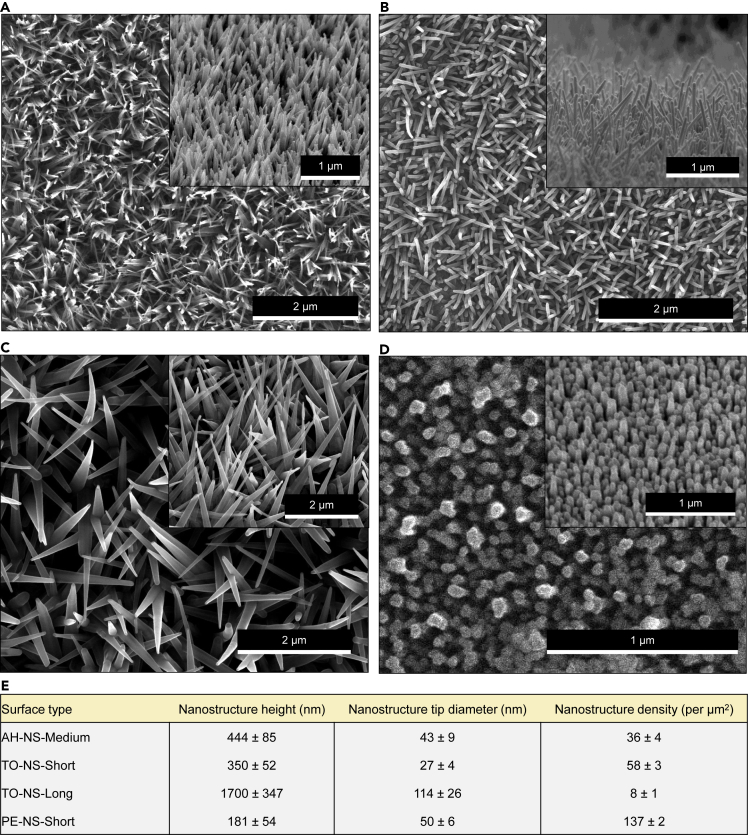
Characterization of TiO_2_ nanostructure surfaces Scanning electron micrographs of AH-NS-medium (A), TO-NS-short (B), TO-NS-long (C), or PE-NS-short (D) surfaces visualized from top view or side view. AH-NS-medium surfaces were generated using the alkaline hydrothermal treatment outlined in Methodology. TO-NS-short surfaces were generated at 715°C for 45 min and 300 standard cubic centimeters per minute (SCCM) flow rate, while TO-NS-long surfaces were generated at 850°C for 45 min and 300 SCCM. PE-NS-short surfaces were generated by plasma reactive ion etching. Average nanostructure height (nm), tip diameter (nm) and density (per μm^2^) for each surface (E) are shown.

Three nanofabrication methods were utilized to generate four nanotopographies that cover a broad range of nanostructure geometries. The nanofabrication methods shown here have previously been used to generate nanotopographies with bactericidal properties, these include alkaline hydrothermal treatment (Cao et al., 2018; Diu et al., 2014), thermal oxidation (Jenkins et al, 2018, 2020; Sjöström et al., 2016), and plasma etching (Dunseath et al., 2019; Hazell et al., 2018b). Alkaline hydrothermal treatment was used to generate titanium dioxide (TiO_2_) nanostructures on commercially pure titanium discs (cpTi), measuring approximately 500 nm in height and ≈50 nm in tip diameter, with a density of 36 per μm^2^. These surfaces are referred to as alkaline hydrothermal nanostructure medium (AH-NS-medium) (Figure 1A). Thermal oxidation was used to generate two different TiO_2_ nanostructure surfaces on grade 5 titanium alloy (Ti-6Al-4V). One surface comprised shorter (350 nm ± 52 nm), sharper (27 nm ± 4 nm), and more dense (58 per μm^2^ ± 3 per μm^2^) nanostructures than AH-NS-medium, herein called thermal oxidation nanostructure short (TO-NS-short) (Figure 1B), while the other comprised much longer nanostructures (1700 nm ± 347 nm), with increased tip diameter (114 nm ± 26 nm) and low density (8 per μm^2^ ± 1 per μm^2^), referred to as thermal oxidation nanostructure long (TO-NS-long) (Figure 1C). Plasma etching was used to generate a nanotopography with the shortest (181 nm ± 26 nm) and most dense (137 per μm^2^ ± 6 per μm^2^) nanostructures on bSi wafers (PE-NS-short) (Figure 1D). SEM was used to quantify the average dimensions and densities of these different nanotopographies (Figure 1E).

### FIB-SEM optimization

**Figure 2:**
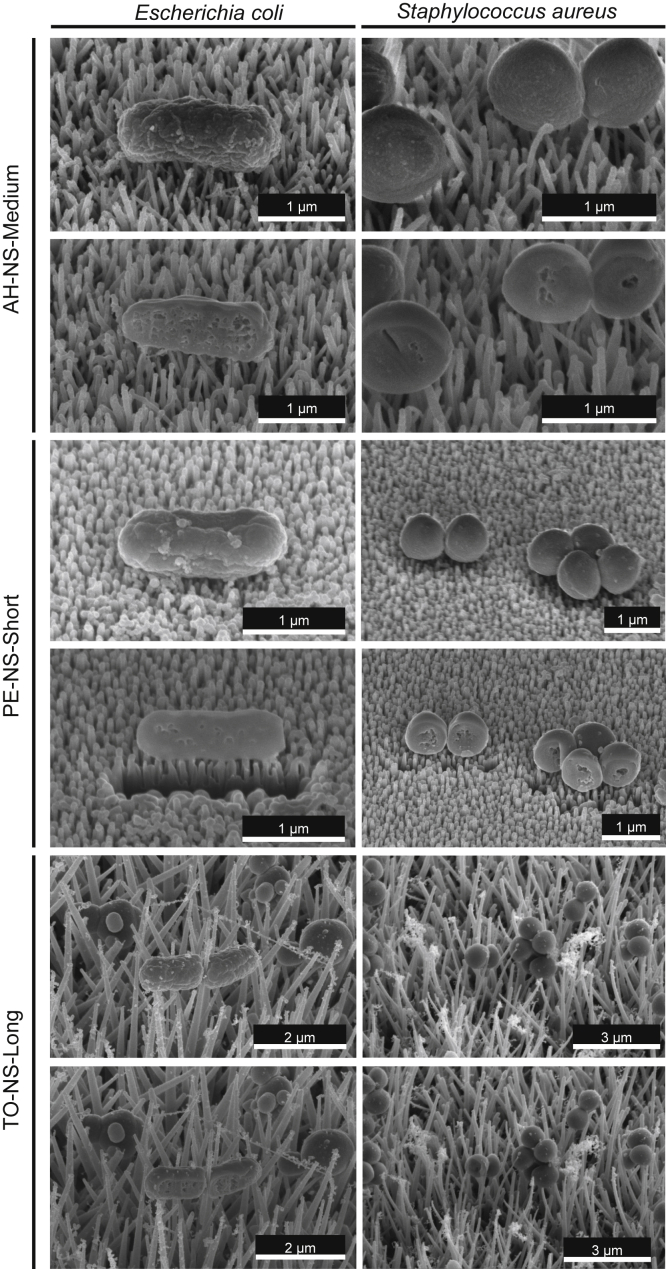
Focused ion beam milling of *E. coli* or *S. aureus* adhered to synthetic nanotopographies Scanning electron micrographs of *E. coli* or *S. aureus* adhered to AH-NS-medium, PE-NS-short, and TO-NS-long, before (upper image) and after (lower image) focused ion beam milling.

To determine sample stability during focused ion beam milling and the extent of beam-induced artifacts introduced, single cross sections were first generated through individual *E. coli* or *S. aureus* cells on different nanotopography types. Generating single cross sections through *E. coli* or *S. aureus* caused minimal movement of bacteria (Figure 2). Consistent with this, generating consecutive cross sections by a slice-by-slice approach produced little sample movement; however, nanostructure charging caused bacteria to move laterally across the field of view on longer nanostructures, resulting in only partial visualization of bacteria-nanotopography interactions (Figure S1). To reduce sample movement during sequential ion beam milling, a protective layer of platinum (0.5 μm in thickness) was deposited on top of each bacterium before slice-by-slice analysis. The addition of platinum greatly reduced bacterial cell drifting during slice-by-slice ion beam milling and minimized curtaining artifacts, providing micrographs with enhanced definition (Figure S2).

### Quantification of bacteria-nanotopography interactions

Area searches of each nanotopography type were performed using SEM to select individual *E. coli* or *S. aureus* cells for analysis by focused ion beam milling. A combination of single cross-sectional analysis and sequential ion beam milling were performed. Slice-by-slice ion beam milling was used to generate consecutive cross sections through selected *E. coli* and *S. aureus* cells. The micrographs collected during sequential cross-sectional analysis were reconstructed into three-dimensional volumes to determine the number of nanostructures in contact with each bacterium. Using these data, a framework was developed to quantify the proportion of nanostructure-induced envelope deformation, penetration, and cell impedence (Jenkins et al., 2020) on a single cell basis. In this study, deformation is defined as the process by which nanostructures directly change bacterial envelope morphology, through indentation. When nanostructures interact with the bacterial envelope with no change in morphology, this is defined as no effect. Penetration is observed when nanostructures peirce through the bacterial envelope, while rupture is defined as penetration combined with a loss of turgor pressure. Furthermore, the effective surface area of bacterial envelope in contact with each nanostructure was determined, and the effect of each interaction on cell morphology and size was investigated.

**Figure 3:**
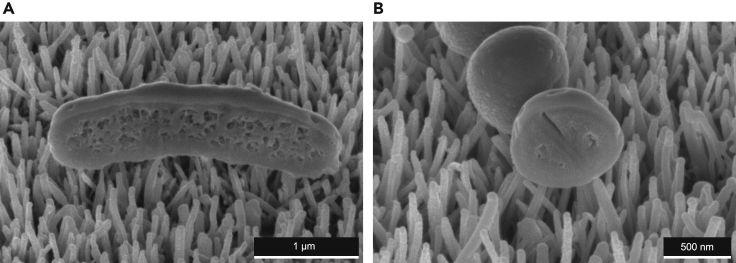
Cross-sectional analysis of *E coli* and *S. aureus* on AH-NS-medium surfaces A single cross section was generated through *E. coli* (A) or *S. aureus* (B) without platinum deposition. The side of *E. coli* in contact with nanostructures is concave, with the mid-cell positioned furthest away from the nanotopography. In contrast, *S. aureus* is positioned on top of the nanotopography with no change in cell shape.

**Figure 4:**
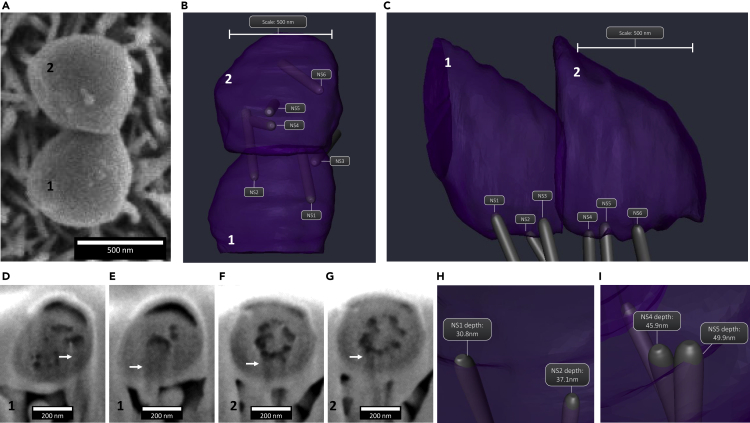
Evidence of nanostructures penetrating and deforming the *S. aureus* cell envelope Top view SEM of two *S. aureus* cells interacting with AH-NS-medium surfaces (A) and 3D reconstruction (B and C). Analysis of *S. aureus* cross section (D) #15 (nanostructure [NS] 1 of cell 1), (E) #25 (NS2 of cell 1), (F) #51 (NS4 of cell 2), and (G) #53 (NS5 of cell 2) showed that a significant portion of each nanostructure had penetrated (white arrows indicate the tip of the nanostructure) into the bacterial envelope by 30.8 nm, 37.1 nm, 45.9 nm and 49.9 nm, respectively. This is clearly shown in the 3D reconstruction (H and I).

On AH-NS-medium surfaces, *E. coli* and *S. aureus* cells predominantly adhered on top of the nanostructures and mainly displayed continuous envelope morphologies with minimal evidence of deformation or penetration. In one example, an *E. coli* cell interacting with nanostructures displayed a concave shape, with the cell midpoint positioned higher above the nanotopography relative to the cellular poles, resulting in very few points of nanostructure contact (Figure 3A). This morphology was not observed for *S. aureus*, leading to more contact points with the nanotopography (Figure 3B). It was noted, however, in one example, that the envelope of *S. aureus* cells on AH-NS-medium surfaces was slightly deformed, which may indicate loss of turgor pressure (Figure 4A). To investigate these interactions in more detail, slice-by-slice ion beam milling of two *S. aureus* cells was performed, and 3D reconstructions were generated to determine whether nanostructures had deformed or penetrated the cell envelope (Figure 4B). Three-dimensional reconstructions revealed three nanostructures interacting with *S. aureus* cell 1 and also for *S. aureus* cell 2 (Figure 4C, Videos S1A and S1B). For *S. aureus* cell 1, two nanostructures (NS1, NS2) had penetrated the envelope (Figures 4D and 4E), reaching depths inside the cell of 30.8 nm and 37.1 nm, respectively (Figure 4H). Two nanostructures (NS4, NS5) had also penetrated the envelope of *S. aureus* cell 2 (Figures 4F and 4G), with nanostructure tips located 45.9 nm and 49.9 nm inside the cell, respectively (Figure 4I). The remaining nanostructures interacting with *S. aureus* cells 1 and 2 (NS3, NS6) had no effect on cell morphology and interacted with 225 nm^2^ and 237 nm^2^ of the cell envelope respectively, representing less than 0.015% of the total cell surface area. Despite multiple nanostructures penetrating the envelope of *S. aureus* cells 1 and 2, there was no evidence of cytosolic leakage, indicating that neither cell lost significant turgor pressure due to nanostructure penetration.

Video S1A. Three-dimensional reconstructions of S. aureus on AH-NS-Medium

Video S1B. Slice and view data processed in Avizo, Related to Figure 4

**Figure 5:**
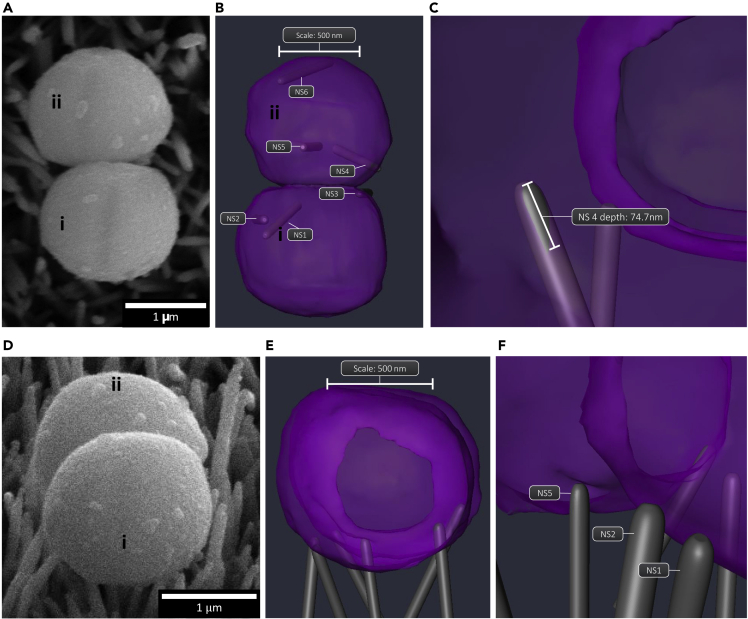
3D FIB-SEM reconstruction of *S. aureus* on AH-NS-medium surfaces SEM micrographs of *S. aureus* cells before automated FIB-SEM cross-sectional analysis was performed (A and D). (B) Six nanostructures directly interacted with two *S. aureus* cells (i and ii). It was found that 3 nanostructures (NS1, 2, 5) caused cell envelope deformation (E and F), while NS4 penetrated the cell by 74.7 nm (C and E).

To determine whether nanostructure-induced envelope penetration occurred in other *S. aureus* cells adhered to AH-NS-medium surface, slice-by-slice ion beam milling was performed on additional *S. aureus* cells with similar envelope morphologies (Figure 5A). Slice-by-slice analysis revealed a total of three nanostructures in contact with *S. aureus* cell 1 and three nanostructures in contact with *S. aureus* cell 2 (Figure 5B, Videos S2A and S2B). For *S. aureus* cell 1, two nanostructures had deformed the envelope (NS1, NS2), interacting with 463 nm^2^ and 147 nm^2^ of the cell envelope, respectively. The remaining nanostructure (NS3) had no effect on envelope morphology, interacting with 248 nm^2^ of the cell envelope. In contrast, the envelope of *S. aureus* cell 2 was penetrated by one nanostructure (NS4) to a depth of 74.7 nm (Figure 5C). A further nanostructure (NS5) had deformed the bacterial envelope and NS6 had no effect on morphology, interacting with 2926 nm^2^ and 155 nm^2^ of the cell envelope, respectively. Consistent with the previous *S. aureus* cell slice-by-slice analysis, envelope penetration did not result in a loss of turgor pressure. In contrast to *S. aureus*, there was no evidence that AH-NS-medium surfaces had penetrated the envelope of *E. coli* and only localized deformation of the cell envelope was observed by generating single cross sections (Figure S3).

Video S2A. Three-dimensional reconstructions of S. aureus on AH-NS-Medium

Video S2B. Slice and view data processed in Avizo, Related to Figure 5

**Figure 6:**
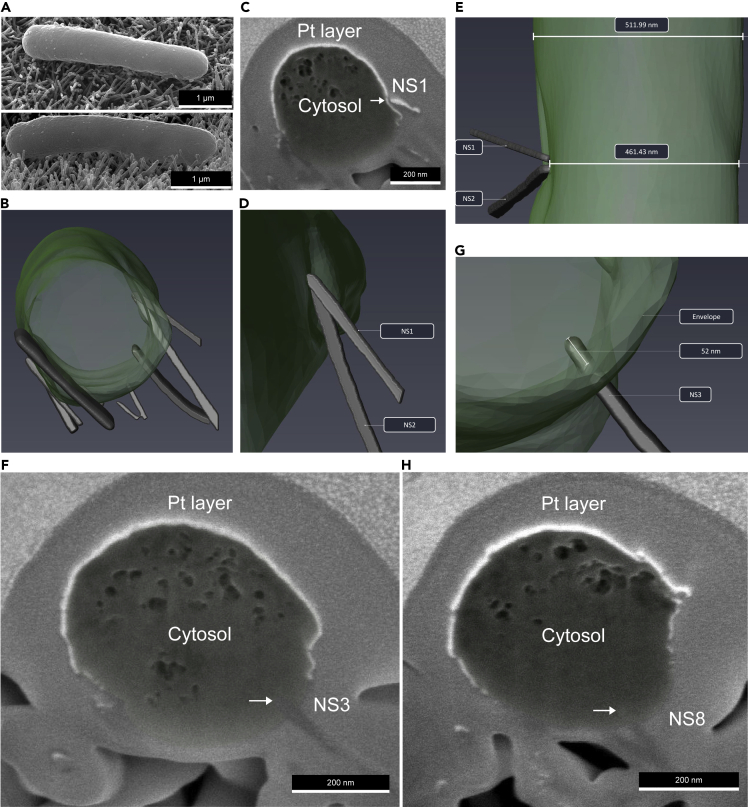
3D FIB-SEM reconstruction of *E. coli* on TO-NS-short surfaces Automated FIB-SEM cross-sectional analysis was performed on an *E. coli* cell (A and B). The focused ion beam produced 80 cross sections (30 nm each) that were imaged and reconstructed in Avizo. Analysis of *E. coli* cross section #32 showed that NS1 had deformed the bacterial envelope without rupture or penetration (C); this is clearly shown in the 3D reconstruction (D and E). Analysis of *E. coli* cross section #42 and #63 showed that NS3 and NS8 had penetrated the bacterial envelope by 52 nm and 37 nm, respectively (F–H).

Nanostructures generated via alkaline hydrothermal treatment (AH-NS-medium) were slightly longer (444 nm ± 85 nm) and wider at the tip (43 nm ± 9 nm) than the nanostructures found on TO-NS-short surfaces, which displayed average lengths of 350 nm ± 52 nm and a tip diameter measuring 27 nm ± 4 nm. Consistent with AH-NS-medium surfaces, *E. coli* cells attached to TO-NS-short surfaces displayed a concave morphology. In one example, the cellular poles of *E. coli* had deformed into the nanotopography, while the mid-cell was suspended above the nanotopography (Figure 6A). Slice-by-slice analysis revealed a total of 8 nanostructures in contact with the cell (Figure 6B, Videos S3A and S3B). Two nanostructures (NS2 and 4) interacted with the side of *E. coli* cell at the same position without penetrating (Figures 6C and 6D), causing the envelope to deform by over 50 nm (Figure 6E). At the cell midpoint, a single nanostructure (NS6) had penetrated the bacterial envelope by 52 nm, without loss of turgor pressure (Figures 6F and 6G). A further nanostructure (NS8) at the cell pole had penetrated the bacterial envelope by 37 nm (Figure 6H). Of note, all eight nanostructures shared a common orientation with respect to the *E. coli* envelope, but only NS6 and NS8 had penetrated the cell, indicating that the point of nanostructure contact along the bottom of the bacterial envelope may be significant in determining the likelihood of penetration. NS2 and NS4 interacted with the side of the *E. coli* cell via the nanostructure tip, causing only envelope deformation. The remaining four nanostructures interacted with the side of *E. coli* but rather than interacting via the nanostructure tips, the side wall of nanostructures formed the point of contact. For *S. aureus,* no evidence of envelope deformation or penetration was observed on TO-NS-short surfaces but in some cases, cells adhered between nanostructures, giving rise to possible cell impedance (Figure S4).

Video S3A. Three-dimensional reconstruction of E. coli on TO-NS-Short

Video S3B. Slice and view data processed in Avizo, Related to Figure 6

**Figure 7:**
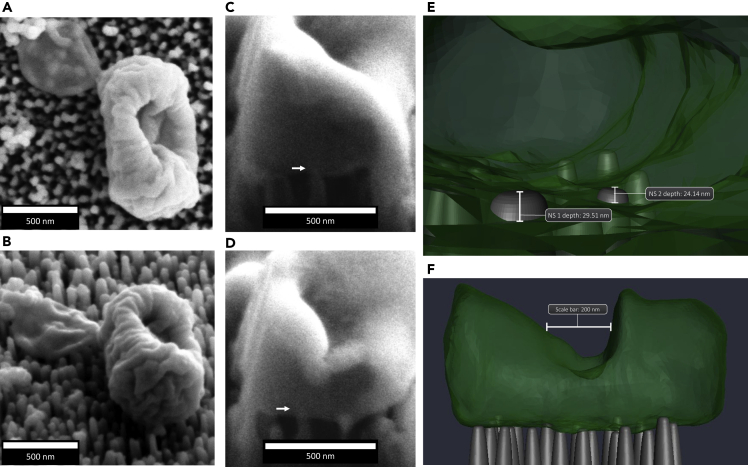
Evidence of potential nanostructure-induced cell impedance of *E. coli* on PE-NS-short surfaces SEM micrographs of *E. coli* cell before FIB-SEM milling (A and B). Cross-sectional analysis of (C) slice #11 (NS1) and (D) slice #18 (NS2) highlights that two nanostructures penetrated the bacterial envelope by 29.5 nm and 24.1 nm, respectively. (E) 3D reconstruction shows the location of the penetrated nanostructures inside the cell, which could have led to the significant change in cell morphology shown in (F).

Similar to TO-NS-short surfaces, the nanotopography of PE-NS-short surfaces consisted of short (181 nm ± 26 nm) and densely packed (137 per μm^2^ ± 6 per μm^2^) nanostructures, measuring 50 nm ± 6 nm in diameter. In contrast to the other nanotopographies, which comprised randomly orientated nanostructures, PE-NS-short nanostructures were aligned in the same vertical direction. Area searches using SEM identified a single *E. coli* cell with significant envelope deformation, synonymous with loss of cytosolic content (Figures 7A–7D). Sequential cross-sectional analysis revealed a total of 24 nanostructures in contact with the *E. coli* cell envelope (Videos 4SA and 4SB). Three-dimensional reconstructions and morphometric analysis revealed that two nanostructures (NS1 and NS2) had penetrated the *E. coli* envelope to depths of 29.51 nm and 24.14 nm, respectively, which may have caused the significant deformation observed (Figures 7E and 7F). The majority of nanostructures (92%) did not penetrate the bacterial envelope, interacting with a total collective surface area of 9000 nm^2^, which corresponds to 0.42% of the total bacterial cell surface area.

Video S4A. Three-dimensional reconstruction of E. coli on PE-NS-Short

Video S4B. Slice and view data processed in Avizo, Related to Figure 7

**Figure 8:**
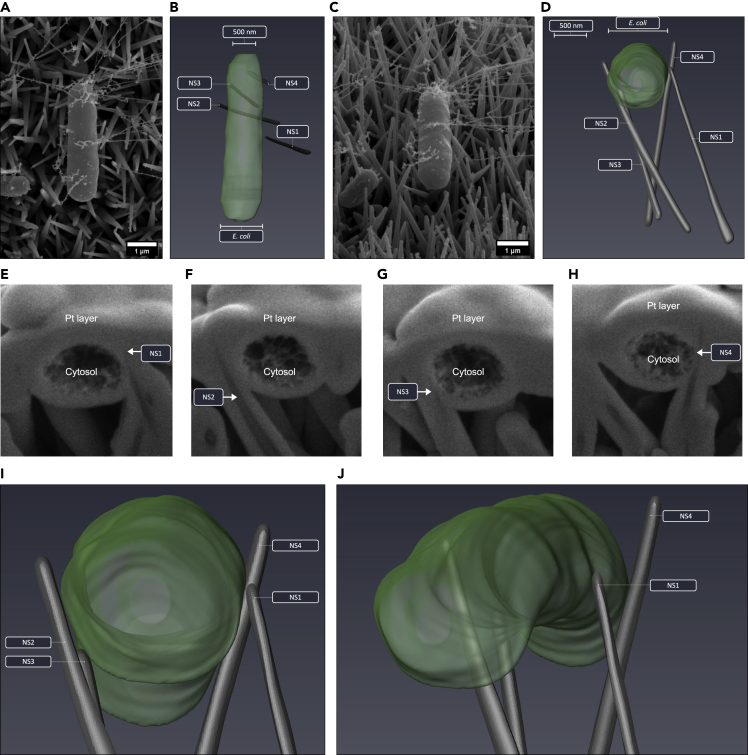
3D FIB-SEM reconstruction of *E coli* impedance on TO-NS-long surfaces Automated FIB-SEM cross-sectional analysis was performed on an *E. coli* cell (A and C) that was pinned between three nanostructures (NS1, 3 and 4) after incubation on a TO-NS-long surface for 3 hr (E–H). There was no evidence of envelope deformation or penetration, and no indication of cytosolic leakage, as the width of the *E. coli* cell remained constant from pole to pole (B, D, I, and J).

**Figure 9:**
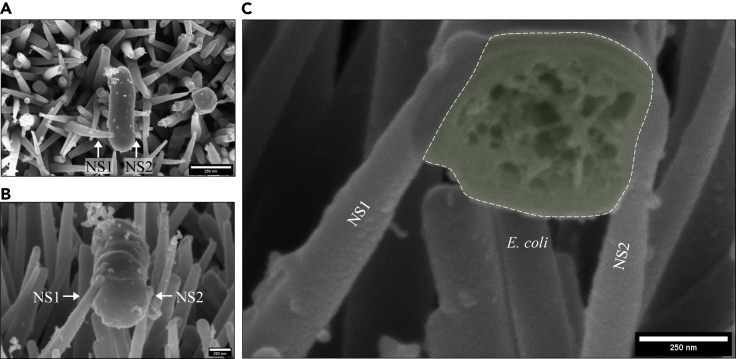
Evidence of potential nanostructure-induced cell impedance on TO-NS-long surfaces SEM micrographs of *E. coli* cell before FIB-SEM milling (A and B). Cross-sectional analysis highlights that *E. coli* is pinned between two nanostructures (NS1-NS2) (C). The cross section through *E. coli* is highlighted in green with a white outline.

In contrast to the other surfaces, for TO-NS-long nanotopographies, *E. coli* and *S. aureus* cells predominantly adhered between adjacent nanostructures, leading to nanostructure-induced cell impedance for both. In one example, an *E. coli* cell expressing significant numbers of fibril-like appendages had adhered between nanostructures (Figures 8A and 8C). Slice-by-slice analysis of the *E. coli* cell identified three nanostructures in direct contact with the side of the *E. coli* cell (Figure 8, Videos 5SA and 5SB). The combined surface area of the three nanostructures in contact with the bacterial envelope was 0.026 μm^2^, collectively interacting with less than 1% of the total cell envelope surface area (14.9 μm^2^). Although none of the nanostructures had penetrated the bacterial envelope, their positioning on either side of the *E. coli* cell could be expected to have acted as a physical barrier that may have prevented cell division. Consistent with this, the dimensions of the *E. coli* cell were highly abnormal, measuring approximately 4 μm in length and 1 μm in diameter (Figure S6). The combination of abnormally large size and absence of cell division septa support our hypothesis of cell impedance and may indicate a nanotopography-induced bacterial stress response, as previously identified (Jenkins et al., 2020). Evidence of nanotopography-induced cell impedance was also observed for *S. aureus* attached to TO-NS-long surfaces. Cross-sectional analysis revealed no evidence of envelope deformation or penetration (Figure S5). In contrast to AH-NS-medium, TO-NS-short, and PE-NS-short surfaces, where the interface between nanotopography and bacteria was primarily formed between the nanostructure tips and the underside of the bacterial cell, for TO-NS-long surfaces, these interactions mostly occurred between the sides of the nanostructures and bacterial cells (Figure 9). In *S. aureus*, the depth of nanostructure penetration varied from 34 nm to 75 nm, while in *E. coli* depths between 27 nm and 45 nm were observed. Additionally, the depth of deformation in the *S. aureus* envelope was 38 nm–64 nm while in *E. coli* deformation from 51 nm to 243 nm was observed. Since these measurments were recorded from different nanotopographies, and different cell numbers, it is unclear whether nanotopography geometries (i.e. density, tip diameter or height) significantly influenced penetration or deformation depth. Additional research on a larger sample size is required to more comprehensively assess this. The quantitative data derived from each 3D model are presented in Table 1.

**Table 1:** Quantitative analyses of bacteria-nanotopography interactions

Bacteria	Nanotopography	Figure	Envelope surface area (μm^2^)	Cell volume (μm^3^)	No. penetrating and %		Depth of penetrating nanostructure (nm)	No. deforming and %		Depth of deformation (nm)	No. impeding and %		Total points of contact	Nanopillar tip contact area (nm^2^)	Contact surface area (%)
*S. aureus* 1	AH-NS-medium	4	1.5	0.14	2	66.66	33.95∗	0	0	–	0	0	3	225	0.01
***S. aureus* 2**	**AH-NS-medium**	**4**	**1.6**	**0.16**	**2**	**66.66**	**47.90∗**	**0**	**0**	**-**	**0**	**0**	**3**	**237**	**0.01**
***S. aureus* 1**	**AH-NS-medium**	**5**	**2.3**	**0.31**	**0**	**0**	**-**	**2**	**66.66**	**38**	**0**	**0**	**3**	**858**	**0.04**
***S. aureus* 2**	**AH-NS-medium**	**5**	**2.1**	**0.28**	**1**	**33.33**	**74.70**	**1**	**33.33**	**64**	**0**	**0**	**3**	**3081**	**0.14**
***E. coli***	**TO-NS-short**	**6**	**5.5**	**0.72**	**2**	**25**	**44.50∗**	**2**	**25**	**51**	**4**	**50**	**8**	**60,958**	**10.60**
***E. coli***	**PE-NS-short**	**7**	**2.3**	**0.23**	**2**	**8**	**26.83∗**	**5**	**21**	**243**	**0**	**0**	**24**	**9001**	**0.43**
***E. coli***	**TO-NS-long**	**8**	**14.9**	**3.35**	**0**	**0**	**-**	**0**	**0**	**-**	**3**	**100**	**3**	**62,630**	**0.18**

Quantitative analyses were performed on each 3D model derived from slice-by-slice FIB-SEM analysis, providing an objective framework from which to derive the possible bactericidal mechanisms of each nanotopography. Definitions for each parameter are indicated below.

Envelope surface area (μm^2^) – the total surface area of bacterial envelope, expressed in μm.^2^.

Cell volume (μm^3^) – the total volume of each bacteria, expressed in μm.^3^.

No. penetrating and % – The number of nanostructures penetrating the bacterial envelope, represented as a single integer and percentage of the total number of nanostructures interacting with the cell.

Depth of penetrating nanostructure (nm) – The depth of nanostructure penetration. Averages were calculated for cells with multiple nanostructure penetrations.

No. deforming and % – The number of nanostructures deforming the bacterial envelope, represented as a single integer and percentage of the total number of nanostructures interacting with the cell.

Depth of deformation (nm) – The depth of envelope deformation. Averages were calculated for cells with multiple envelope deformations.

No. impeding and % – The number of nanostructures impeding the bacterial envelope, represented as a single integer and percentage of the total number of nanostructures interacting with the cell.

Total points of contact – The total number of bacteria-nanotopography interactions.

Nanopillar tip contact area (μm^2^) – The total surface area of nanopillar interacting with the bacterial envelope, expressed in μm^2^. Calculated by summing individual nanopillar tip contact areas.

Contact surface area (%) – The proportion of the bacterial cell envelope interacting with nanostructure tips, expressed as a percentage of the total bacterial envelope surface area.

Video S5A. Three-dimensional reconstruction of E. coli on TO-NS-Long

Video S5B. Slice and view data processed in Avizo, Related to Figure 8

## Discussion

It is generally accepted that the antibacterial activity of natural and synthetic nanotopographies is driven by physical contact with nanostructures (i.e. nanowires, nanopillars, nanocones, nanospikes, nanospears). This can result in penetration or rupture of the bacterial cell envelope, or damage can be inflicted via cell impedance or induction of oxidative stress responses (Jenkins et al., 2020; Linklater et al., 2021; Tripathy et al., 2017). Visualizing the adhesion interface between bacteria and nanotopographies is thus of critical importance for determining by which mechanisms they mediate their antibacterial effects. In this study, we utilized an FIB-SEM method for directly viewing, in three-dimensional space, physical interactions between the cell envelope of *S. aureus* or *E. coli* and four nanotopographies of different geometries (AH-NS-medium, PE-NS-short, TO-NS-short or TO-NS-long). Morphometric analysis was performed to quantify these interactions. The first published morphometric analysis of 3D volume reconstructions generated by FIB-SEM was of brain cells (Jorstad et al., 2015). In this study, Jorstad *et al.* recognized that a gap exists between rapid 3D volume reconstruction techniques, such as slice-by-slice FIB milling, and software for model quantification; analyses that have previously been achieved via manual segmentation methods. Similarly, generating a 3D model of bacteria interacting with nanostructured surfaces may provide additional qualitative insights, but does not directly provide morphometric data. Therefore, we utilized NeuroMorph software package to quantify morphometric parameters of bacteria-nanotopography interactions, including the intrinsic contact area of the nanostructured surface with the bacterial cell envelope and the bacterial cell volume.

The number of bacteria-nanotopography interactions correlated with nanotopography density, with PE-NS-short surfaces displaying the highest number of physical points of contact (24 interactions per cell) and TO-NS-long nanotopographies having the lowest (3 interactions per cell). However, number of contact points did not correspond to frequency of nanostructure-induced envelope penetration. Rather, nanotopographies with reduced nanostructure density (TO-NS-short and AH-NS-medium) exhibited higher levels of nanostructure-induced envelope penetration (25% and 66%, respectively) compared with PE-NS-short (8%). One possible explanation for this could be the relative surface area of bacterial envelope that nanostructures interact with simultaneously. From the bacteria analyzed in this study, nanostructures on PE-NS-short surfaces interacted with <1% of the total bacterial surface area, while on AH-NS-medium and TO-NS-short nanotopographies, the surface area of physical contact was 3.5–22 times greater. It is also possible that these differences were influenced by nanostructure orientation and/or the simultaneous variation in height or tip diameter between nanotopographies, which affects the precise contact point with the bacterial envelope and forces exerted. Although nanostructure tip diameter is generally greater on PE-NS-short surfaces compared with AH-NS-medium and TO-NS-short surfaces, the nanostructures have the same orientation and a much higher density, meaning that bacteria-nanotopography interactions will be mediated by the nanostructure tips. In contrast, nanostructure orientation on all the other surface types was random, giving rise to bacteria-nanotopography interactions that were mediated by nanostructure tips and/or nanostructure sidewalls.

In this study, analysis of three-dimensional reconstructions of *E. coli* on PE-NS-short surfaces revealed nanostructure-mediated envelope penetration and significant loss of turgor pressure, indicating that the cell wall may have been ruptured, as predicted by the biophysical model (Li, 2015; Pogodin et al., 2013; Xue et al., 2015). However, due to the resolution limit of FIB-SEM, no qualitative evidence of cell wall rupturing was identified. Evidence of nanostructure-mediated envelope penetration was also observed on AH-NW-short and TO-NS-short nanotopographies, but this did not result in cell rupture or loss of turgor pressure. One possible explanation for this observation could be the increased nanostructure density on PE-NS-short surfaces, which would lead to more points of contact with the bacterial envelope. Combined with envelope penetration, this could result in the cell rupturing. Current dogma infers that bacterial cells rupture interstitially between nanopillars (Ivanova et al., 2012). Rather, our observations suggest that cumulative levels of envelope penetration or simply deformation localized at the bacterium-nanostructure tip interface could be a principal driver of physical damage and subsequent antibacterial activity. This is consistent with our previous studies (Jenkins et al., 2020) and is supported by recent modeling that indicates that envelope deformation around nanopillar tips delivers sufficient in-plane strain to locally damage and penetrate bacteria (Velic et al., 2021). It is also possible that the biophysical model is only applicable to cicada wing-like nanotopographies such as PE-NS-short, where nanostructure height, spacing, and diameter are more uniform across the surface, whereas dragonfly wing-like nanotopographies, including AH-NS-medium, TO-NS-short and TO-NS-long, display uneven distribution of height, density, and tip diameter. Thus, stretching and rupturing of the suspended bacterial cell wall may be unlikely on surfaces comprising nanostructures of random height and orientation.

Morphometric analysis of the 3D volume reconstructions revealed a strong correlation between cell impedance and cell dimensions. Cell impedance was observed for *S. aureus* cells incubated on TO-NS-short surfaces but not for *E. coli*. One possible explanation for these differences is the size and shape of *E. coli* relative to *S. aureus.* Based on the longer, elongated shape and larger surface area of *E. coli* cells, it is more likely for adhesion to occur on top of the nanostructures rather than in-between. Furthermore, nanotopography density influenced the likelihood of cell impedance, since *E. coli* cells were mostly found to adhere between nanostructures on TO-NS-long surfaces, where nanostructure spacing was generally greater than the width of *E. coli* cells (≈500 nm). In contrast, the smaller cell diameter and coccoid morphology of *S. aureus* increased the likelihood of attachment between nanostructures, irrespective of surface type. These findings are consistent with previous literature investigating the effects of microtopography on microbial retention. Titanium surfaces with 0.5 μm–2 μm pit sizes were found to retain significantly more *S. aureus* cells compared to *P. aeruginosa* or *Candida albicans* following 1 hr incubation, owing to the smaller diameter of *S. aureus* (Whitehead et al., 2005). A similar mechanism was recently observed on titanium nanostructure surfaces with pocket-like formations. *S. epidermidis* cells were found to settle inside the pockets, limiting biofilm growth (Cao et al., 2018). Considering these findings, it is reasonable to hypothesize that cell impedance would occur more frequently with smaller bacterial cells, such as *S. aureus,* according to the relative dimensions of bacteria and nanostructure. Other factors, including cell surface charge and hydrophobicity, may have also influenced bacterial adhesion to nanostructured surfaces (Krasowska and Sigler, 2014).

The FIB-SEM method presented in this study enabled physical contact points between bacteria and nanotopography to be visualized. This method was applied to a range of nanotopographies of varying nanostructure geometries and composition. The staining protocol used here enabled the envelope of *S. aureus* and *E. coli* to be resolved with nanometer resolution and was clearly distinguishable from the bacterial cytosol. A further advantage of this approach was that cross sections could be generated through bacteria at any desired location, enabling all points of contact between bacteria and nanotopography to be resolved. Additionally, by performing sequential slice-by-slice ion beam milling, three-dimensional volume reconstructions could be generated to allow 360° visualization and quantification of the morphometric information. The data generated from these analyses demonstrate how a framework for quantifying bacteria-nanostructure interface interactions can be developed to better assess the antibacterial mechanisms of nanotopographies. We anticipate that the FIB-SEM approach highlighted in this study could be widely used to progress our mechanistic understanding of nanotopography-mediated antibacterial activity.

### Limitations of the study

The cross-sectional analyses of *E. coli* and *S. aureus* in contact with nanostructured surfaces performed in this study are representative of a single time point (3-hr surface incubation). This study explored two bacterial species only, future research should include a greater variety of microorganism in both planktonic and biofilm phases of growth. Owing to cost and limited access to FIB-SEM equipment, and long data collection times for each bacteria, the analyses and conclusions in this study are representative of a small sample size of seven bacteria, which the authors recognize is a limitation. Additional access to FIB-SEM equipment is required to assess the morphological changes more comprehensively in Gram-negative and Gram-positive bacteria over a broader time range.

## STAR★Methods

### Key resources table

**Table:** 

REAGENT or RESOURCE	SOURCE	IDENTIFIER
**Bacterial and virus strains**		

*Escherichia coli* K12	Provided by A. Edwards (UB2568)	Blattner et al. (1997)
*Staphylococcus aureus* Newman	Provided by T. Foster (UB1621)	Baba et al. (2008)

**Chemicals, peptides, and recombinant proteins**		

Luria Bertani (LB) broth	BD Biosciences	N/A
Ethanol (99.99%)	Fisher Scientific	N/A
Glutaraldehyde (EM grade)	Fisher Scientific	N/A
Osmium tetraoxide	Agar Scientific Ltd. Essex, UK	N/A
Osmium	Agar Scientific Ltd. Essex, UK	N/A
Sodium cacodylate	Sigma-Aldrich, St. Louis, USA	N/A
Thiocarbohydrazide	Sigma-Aldrich, St. Louis, USA	N/A
Potassium ferrocyanide	Sigma-Aldrich, St. Louis, USA	N/A
Sodium hydroxide	Fisher Scientific	N/A
Colloidal silver paste	Agar Scientific Ltd. Essex, UK	N/A

**Software and algorithms**		

Blender	V2.9.0	https://www.blender.org
Avizo	V9.7.0	https://www.thermofisher.com/ca/en/home/industrial/electron-microscopy/electron-microscopy-instruments-workflow-solutions/3d-visualization-analysis-software/avizo-materials-science.html
Microsoft Paint 3D	V6.21	N/A
NeuroMorph		https://github.com/NeuroMorph-EPFL/NeuroMorph
AutoDesk	V2020	https://www.autodesk.co.uk/products/autocad/overview?term=1-YEAR
Excel	V16.49	Microsoft

**Other**		

Ti-6Al-4V Grade 5 titanium alloy	Titanium Metals LTD	N/A
Silicon carbide (SiC) grit papers	Struers	N/A
Polisher	Struers TegraForce1	N/A
Tube furnace	Elite Thermal Systems LTD	N/A
Commercially pure titanium	Ti-Tek (UK) LTD	N/A
Reactive ion etching system	Oxford Instruments	N/A
Oven	Gallenkamp Plus II	N/A
Critical point dryer	Leica CPD300	N/A
PTFE holders	This study	N/A

### Resource availability

#### Lead contact

Further information and requests for resources should be directed to and will be fulfilled by the lead contact, Bo Su, University of Bristol, United Kingdom (b.su@bristol.ac.uk)

#### Materials availability

This study did not generate new unique reagents.

#### Data and code availability

All data produced or analyzed for this study are included in the published article and its supplementary information file. Any additional information required is available from the lead contact upon request. This paper does not report original code.

### Experimental model and subject details

#### Bacterial strains and culture conditions

*E. coli* K12 (Blattner et al., 1997) and *S. aureus* Newman (Baba et al., 2008) were used in this study. Broth cultures were incubated for 16 h at 37°C, 220 rpm, subcultured to OD_600_ 0.1 and grown to mid-exponential phase. Bacteria were cultured in Luria Bertani (LB) broth (BD Biosciences). Titanium samples were sterilized in absolute ethanol, washed in dH_2_O and dried prior to inoculation with bacterial suspensions. All surfaces were inoculated with 50 μL of bacterial suspension (10^6^-10^7^ colony forming units (CFU)), forming a meniscus, and were incubated statically at 37°C for 3 hours.

### Method details

#### Thermal oxidation

TiO_2_ nanostructure surfaces were generated using a thermal oxidation procedure previously outlined (Jenkins et al., 2020). The exact methodology used is as follows: Grade 5 titanium alloy (Ti-6Al4V, Titanium Metals Ltd) samples (0.64 cm^2^) were machine polished (Struers® TegraForce1) using decreasing silicon carbide grit sizes (#80, #500, #1200, #2000, and #4000). To remove surface contaminants, titanium discs were placed inside a digital ultrasonic bath (Grant Scientific XUB series) in distilled water (dH_2_O), and the samples were cleaned at 40°C for 15 minutes using 100% power. Following ultrasonication, titanium samples were placed in ethanol (analytical reagent grade (99.99%), Fisher Scientific) for 10 minutes before air drying. Polished and cleaned titanium samples were sealed inside a horizontal alumina tube (120 cm x 11 cm outer x 9 cm inner) positioned in a furnace (Elite Thermal Systems Ltd). Prior to thermal oxidation, the furnace was purged with inert argon gas (Ar) to remove oxygen and achieve a one-directional flow. Following purging, heating was initiated at 15°C/minute until a predefined maximum was reached; in this study, temperatures of 715°C and 850°C were used. Once the final temperature was reached, Ar was redirected into a sealed Duran^TM^ vessel containing liquid acetone (analytical reagent grade (99.99%), Fisher Scientific), maintained at 25°C. This generated an acetone uvapor phase, which initiates the oxidation reaction with titanium samples. Following completion of the heating programme, furnace cooling began. The flow of Ar was maintained at a constant rate until room temperature was reached.

#### Alkaline hydrothermal treatment

Commercially pure titanium (Ti-Tek (UK) Ltd) with 0.7 mm thickness was laser cut into 11 mm circular disks by Laserit. All disks were mirror polished (TegraPol-15, Struers) before being washed with deionized water and alcohol for 10 minutes each. The disks were air dried and slotted into custom-made PTFE holders to keep the disks upright and placed into a 125 ml PTFE acid-digestion vessel containing 1 M NaOH (52 ml). The vessel was tightly sealed and placed in a preheated oven (Gallenkamp Plus II) for 2 hours at 250^o^C. After the hydrothermal treatment, the disks were cooled, washed with deionized water and absolute ethanol, dried and treated at 300^o^C for 1 h before immersion in 0.6 M HCl for exchanging the sodium ions with hydrogen ions. The disks were rinsed with copious amounts of deionized water and placed in a furnace for 2 h at 600^o^C.

#### Plasma etching

Plasma etching of n-doped single-crystal silicon (100) wafers was performed in Oxford Instruments reactive ion etching (RIE) systems fitted with inductively coupled plasma (ICP) sources. ICP etching of a Si wafer was used to generate cicada wing-inspired, short nanopillars (∼0.2 μm in length).

#### FIB-SEM sample preparation

Samples were prepared using previously described methods (Jenkins et al., 2020). The exact methodology used is as followed: Following overnight fixation in 2.5% EM grade glutaraldehyde at 4°C, samples were washed (3 x 5 minutes) in 0.1 M sodium cacodylate buffer prior to OTO (Osmium tetraoxide – Thiocarbohydrazide – Osmium) processing. Briefly, this method included post fixation in equal volumes of 4% osmium tetraoxide (Agar Scientific Ltd. Essex, UK) and 3% potassium ferrocyanide (Sigma-Aldrich, St. Louis, MO, USA) for 60 minutes on ice. Following post fixation, samples were rinsed (3 x 5 minutes) in dH_2_O before incubating with thiocarbohydrazide (Sigma-Aldrich, St. Louis, MO, USA) for 20 minutes. Additional dH_2_O washing steps (3 x 5 minutes) were applied before incubation in 2% aqueous osmium for 30 minutes at room temperature. Following OTO processing, bacterial samples were stained in 1% aqueous uranyl acetate (1 hour at 4°C) followed by lead aspartate (200 μM) for 30 minutes in the dark. Between these steps, washing with dH_2_O was performed. After the final washing step, bacterial samples were dehydrated in a graded ethanol series of 25%, 50%, 70%, 90% and 100% (Sigma-Aldrich, St. Louis, MO, USA). Samples were then critically point dried using a Leica CPD300, following an established protocol for microbial cells(LiYu et al., 2014). Titanium discs were mounted onto 0.5” aluminum stubs using colloidal silver paste (Agar Scientific Ltd. Essex, UK), before being coated with a 10 nm chromium layer using an Emitech K757X sputter coater system.

#### Sequential ion beam milling

Two microscopes were used to perform ion beam milling: 1) Strata FIB201 (University of Manchester); 2) FEI Scios (DESY NanoLab). Samples were loaded into the chamber and the system was purged to create a vacuum. Before cross-sectional analysis, the stage was tilted by 52°, moving the titanium discs perpendicular to the gallium ion beam. Area scans were performed at an accelerating voltage of 5 kV and current of 50 pA. Prior to ion beam milling, a protective platinum layer (500 nm) was deposited onto each bacterium. Rough cut trenches were milled around coated bacteria to depths of 250 nm using an accelerating voltage of 30 kV and current of 1 nA. Auto Slice and View software was used to carry out sequential sectioning of *E. coli* in 30 nm slices and 20 nm for *S. aureus* cells. This was performed with an accelerating voltage of 30 kV and beam current of 30 pA. Images of each section were acquired using electron beam accelerating voltages of 5 kV and current of 50 pA.

#### FIB-SEM image processing and 3D volume reconstruction

Slice and view data were processed using previously described methods (Jenkins et al., 2020). The exact methodology used is as follows: The slice and view data acquired from sequential FIB milling was processed using the FIB-stack wizard tool in Avizo v9.7.0 (FEI). Briefly, this tool facilitates aligning the FIB-stack and correction of geometrical artefacts such as stage tilt foreshortening and/or vertical shift. Avizo segmentation editor was utilized to reconstruct 3D volumes of bacteria and to visualize interactions with all nanostructures.

### Quantification and statistical analysis

#### Morphometric analysis of 3D models

Morphometric analysis of the 3D models was performed using NeuroMorph add-on in Blender (V2.9.0) 3D modeling software, as detailed previously (Jorstad et al., 2015). Briefly, the 3D models reconstructed in Avizo V9.7.0 software were exported as .obj files, which can be used in other 3D modeling software such as Blender, Microsoft Paint 3D or AutoDesk suite. The exported 3D models were then imported and the morphometric analysis was performed inside Blender by using NeuroMorph. Quantification of nanotopographies and bacteria:nanotopography interactions was performed using Microsoft Excel.

### Additional resources

Our study has not generated or contributed to a new website/forum and it is not part of a clinical trial.
