# Supplementary material for: Resolving physical interactions between bacteria and nanotopographies with focused ion beam scanning electron microscopy
Source: iScience. 2021 Jul 7;24(7):102818. doi: 10.1016/j.isci.2021.102818 (PMC8319809; doi:10.1016/j.isci.2021.102818)
Supplement: Document S1. Figures S1–S6 [file mmc1.pdf]

iScience, Volume *24*

Supplemental information

Resolving physical interactions between bacteria and nanotopographies with focused ion beam scanning electron microscopy

Joshua Jenkins, Mohd I. Ishak, Marcus Eales, Ali Gholinia, Satishkumar Kulkarni, Thomas F. Keller, Paul W. May, Angela H. Nobbs, and Bo Su

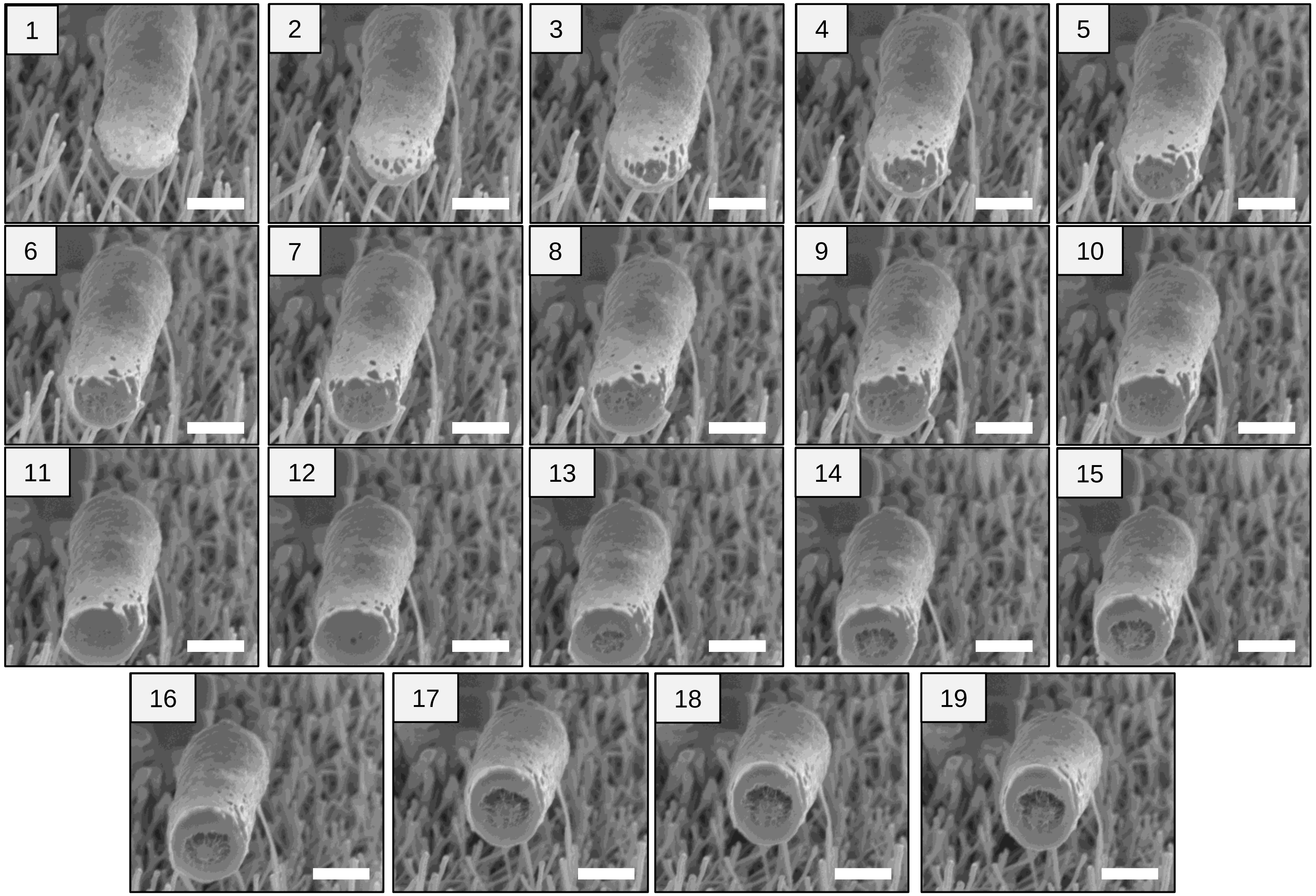

Supplementary Figure 1. Slice and view focused ion beam milling of *E. coli* without platinum deposition. FIB-SEM was used to generate sequential 30 nm cross sections through *E. coli* without a protective platinum coating. The milling interface of 19 serial sections is shown. Scale bar: 500 nm. Related to Figure 2.

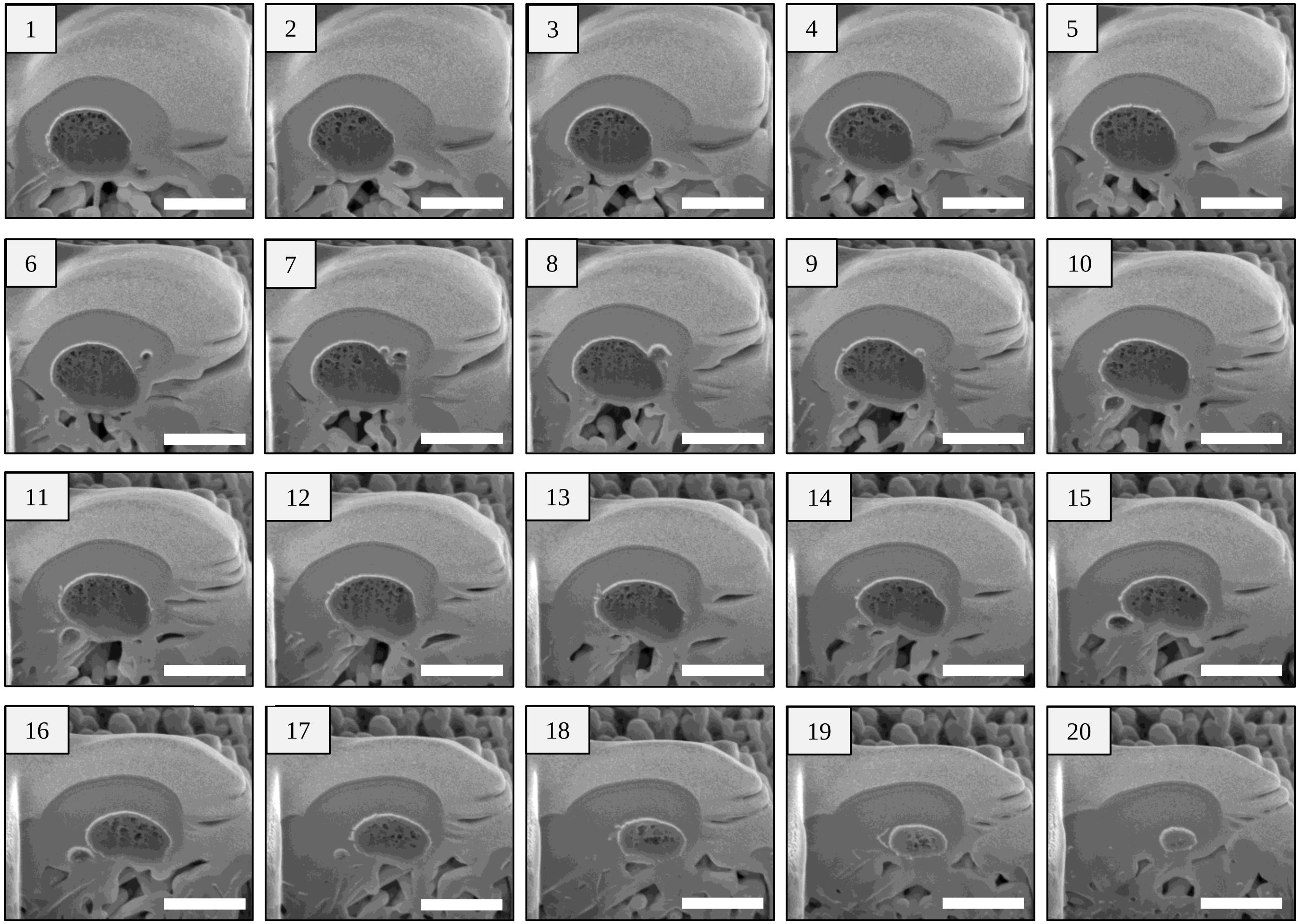

Supplementary Figure 2. Slice and view focused ion beam milling of *E. coli* with platinum deposition. FIB-SEM was used to generate sequential 30 nm cross sections through *E. coli* with a protective platinum coating. The milling interface of 20 serial sections is shown. Scale bar: 500 nm. Related to Figure 2.

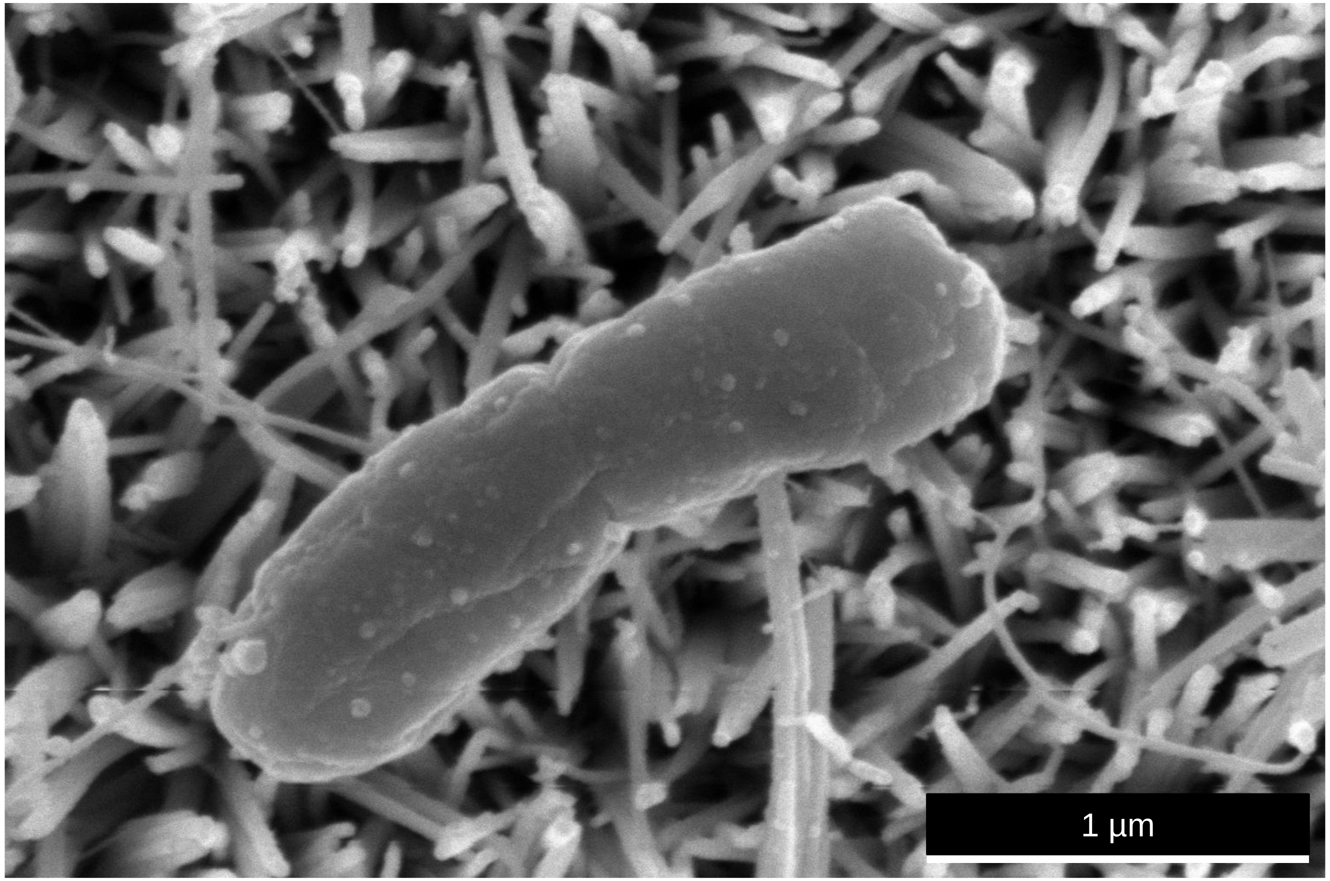

Supplementary Figure 3. ***E. coli*** **envelope deformation induced by AH-NS-Medium surface.** No evidence of envelope penetration was observed for *E. coli* on AH-NS-Medium surfaces. However, many *E. coli* cells displayed envelope deformation at the point of nanostructure contact, as represented in this micrograph. Related to Figures 3, 4 and 5.

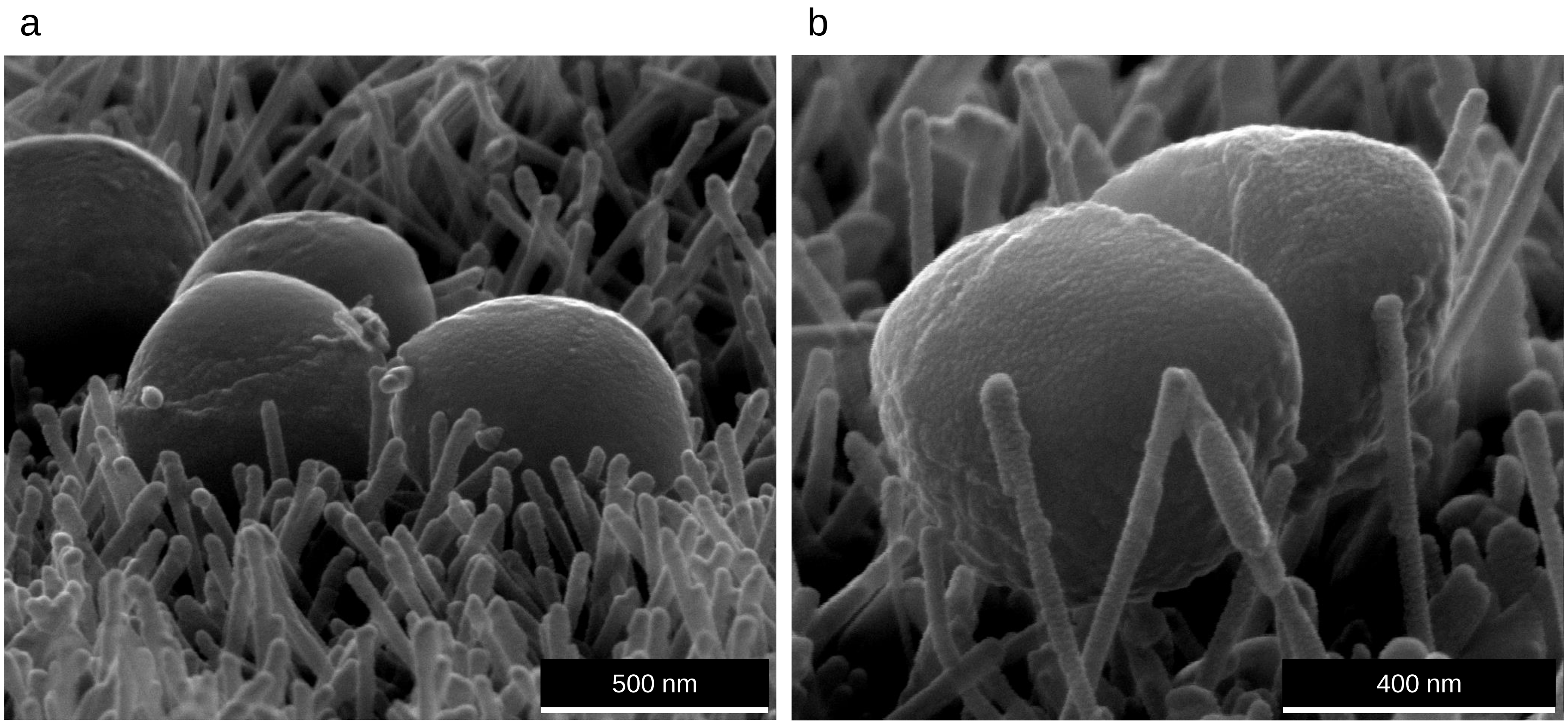

Supplementary Figure 4. ***S. aureus*** **cell impedance induced by TO-NS-Short surface.** Representative scanning electron micrographs of *S. aureus* cells impeded by TiO_2 nanostructures on TO-NS-Short surfaces (a-b). The random growth direction of TiO_2 nanostructures on TO-NS-Short surfaces generates variation in nanostructure density across the surface, meaning that some *S. aureus* cells adhere between nanostructures that are spaced greater than 500 nm apart. Related to Figure 6.

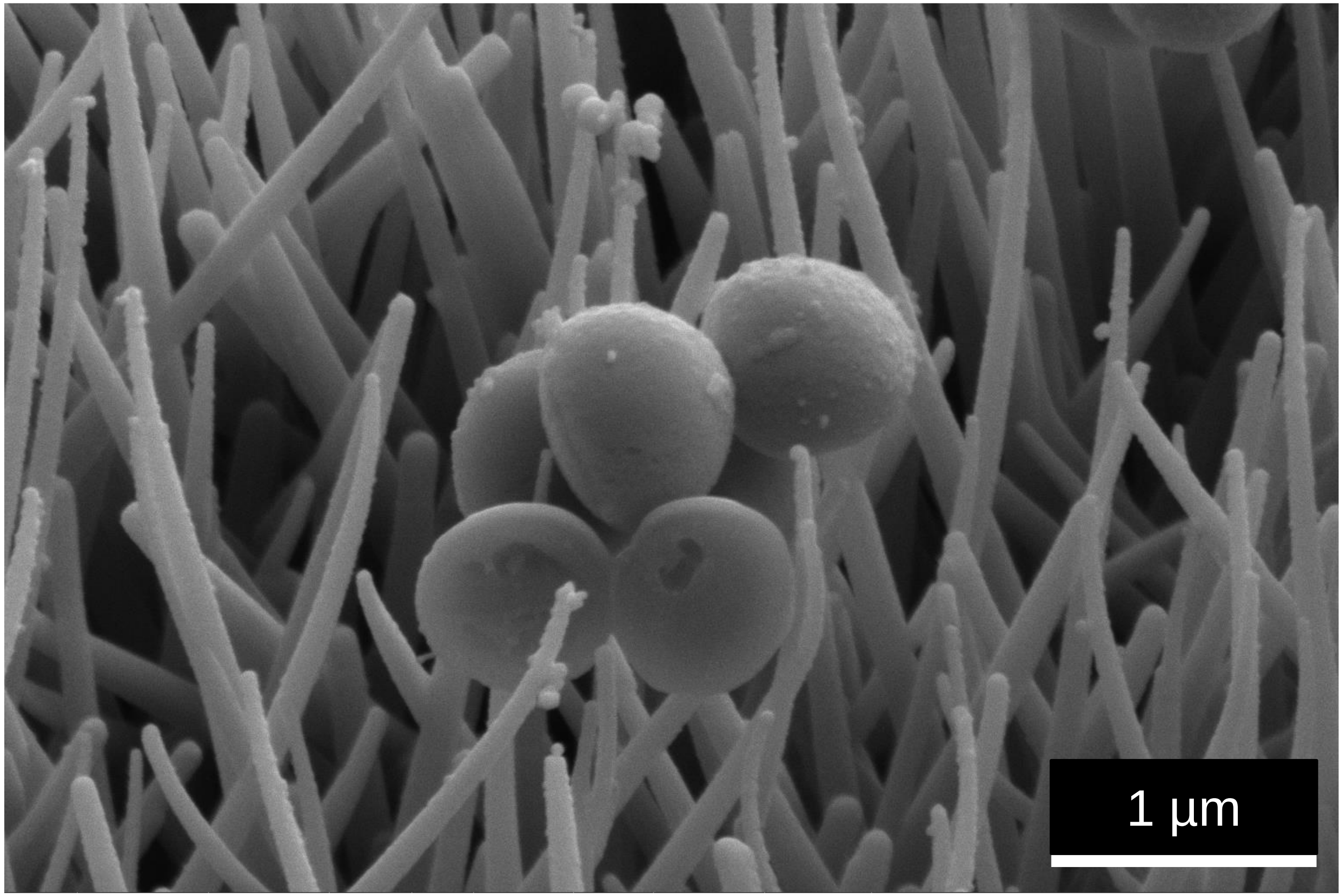

Supplementary Figure 5. Evidence for *S. aureus* cell impedance induced by TO-NS-Long surface. Representative scanning electron micrographs of *S. aureus* impeded by TiO_2 nanostructures on TO-NS-Long surfaces. Cross sections through *S. aureus* cells show no evidence of envelope deformation or penetration, but clearly show that *S. aureus* cells have adhered between the nanostructures. Related to Figures 8-9.

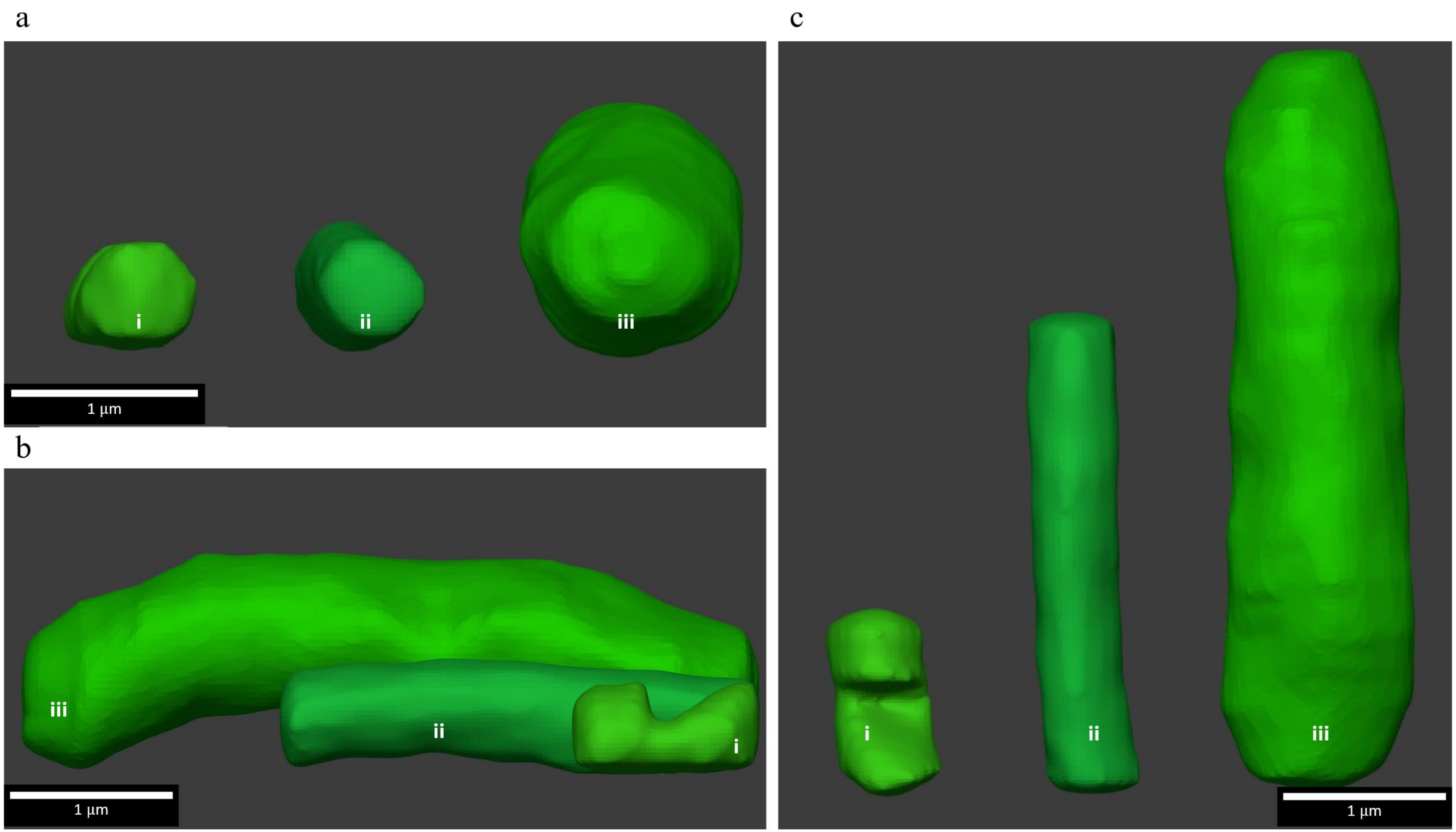

Supplementary Figure 6. **Size comparison of *E. coli* cells adhered to different nanostructured surfaces.** Size comparison of three different *E. coli* cells adhered on (i) PE-NS-Short surface, (ii) TO-NS-Short surface and (iii) TO-NS-Long surface. (a) Front view, (b) side view, (c) top view. Related to Figures 6-8.
